# Supplementary material for: Recreating the biological steps of viral infection on a cell-free bioelectronic platform to profile viral variants of concern
Source: Nat Commun. 2024 Jul 3;15:5606. doi: 10.1038/s41467-024-49415-6 (PMC11222515; doi:10.1038/s41467-024-49415-6)
Supplement: Supplementary file 1 — Supplementary Information [file 41467_2024_49415_MOESM1_ESM.docx]

Supplementary Materials for

Recreating the Biological Steps of Viral Infection on a Cell-free Bioelectronic Platform to Profile Viral Variants of Concern

*Zhongmou Chao,^1†^ Ekaterina Selivanovitch,^1†^ Konstantinos Kallitsis,^2^ Zixuan Lu,^2^ Ambika Pachaury,^1^ Róisín Owens,^2^ Susan Daniel^1^**

* Corresponding author. Email: sd386@cornell.edu

**Contents:**

1. Figure S1. Validation of Spike incorporation into SARS-CoV-2 pseudoparticles and ACE2 and TMPRSS2 in cells using Western Blots.
2. Figure S2. Cell bleb characterization using NanoSight Nanoparticle Tracking Analyzer.
3. Figure S3. Cell bleb size as a function of time obtained from Dynamic Light Scattering over 21 days.
4. Figure S4. FRAP data collected from hybrid SLBs assembled using Vero E6 cell-derived blebs, done in triplicate with recovery curves for each respective sample.
5. Figure S5. FRAP data collected from hybrid SLBs assembled using Vero E6 TMPRSS2 cell-derived blebs, done in triplicate with recovery curves for each respective sample.
6. Figure S6. Intensity line profiles of images acquired during FRAP experiments.
7. Figure S7. Early entry pathway recapitulated on host cell (+ACE2, +TMPRSS2) hybrid SLB and appropriate controls.
8. Figure S8. VPP_Spike_ interactions with hybrid SLBs with (**a**) and without (**b**) ACE2 receptors.
9. Table S1. A summary of all fitted hybrid SLB electrical properties reported in Figs 3-5.
10. Figure S9. Late entry pathway recapitulated on host cell (+ACE2) hybrid SLB.
11. Figure S10. FRAP data collected from hybrid SLBs assembled using wtSpike-transfected HEK 293T cell-derived blebs.
12. Table S2. Sequences for the three Spike variants used for this study, including Wuhan-Hu-1 Spike, Omicron BA.1, and Omicron BA.4, Gag-Pol, and Luciferase.
13. Figure S11. Optimization of R18 labeling of SARS-CoV-2 pseudoparticles to achieve semi-quenched labeling state for optical imaging of fusion.
14. Figure S12. The impact of ethylene glycol and acidic PBS buffer on PEDOT:PSS electrode electrical properties.
15. **Validation of Spike protein, ACE2, and TMPRSS2 using Western Blots**

**Figure S1.** Validation of Spike incorporation into SARS-CoV-2 pseudoparticles and ACE2 and TMPRSS2 in cells using Western Blots. (**a**) Representative western blot of pseudoparticles (VPPs) containing Spike (Wuhan-Hu-1). Pseudoparticles were resolved using gel electrophoresis, transferred, and then stained to visualize the SARS-CoV-2 S2 domain using a Spike rabbit polyclonal antibody (Sino Biological). (**b**) Representative western blot of the ladder (lane 1) and pseudoparticles containing Omicron BA.1 (lane 2) and Omicron BA.4, (lane 3). Lane 4 contains blebs derived from HEK 293T cells transfected with wtSpike (Wuhan-Hu-1), which were used in our reverse configuration experiments where the SLB modeled the virus surface, while the blebs represented the cell surface. All the variant VPPs in (b) contained both cleaved (S2) and uncleaved (S0) Spike constructs, which potentially contributed to the loss in fusogenicity we observed on our platform compared to the wtSpike VPPs (a). A chemiluminescent western blot detection method was applied, using HRP-conjugated antibody coupled with enhanced chemiluminescent substrates. As this method is semi-quantitative, these western blots show only the presence or absence of a protein. To analyze the cell surface expression of receptor (**c**) ACE2 (top) and protease TMPRSS2 (bottom) in VeroE6 (ATCC # CRL-1586) and Vero/TMPRSS2 (JCRB # 1818) cell lines, cells were treated with 400 μl of biotin buffer (250 μg/ml ThermoFisher Sulfo-NHS-SS-Biotin in PBS) to label the surface proteins with biotin. Afterwards 50 mM glycine in PBS was then added for 30 min to the cells. Subsequently, the cells were lysed using a lysis buffer (0.1% TritonX in 1X TBS, 1 Complete Protease Inhibitor tablet from Sigma) and centrifuged at 14,000 rpm for 10 min at 4˚C. The supernatant was then added to 40 μl of Streptavidin beads (Pierce Thermofisher). Overnight incubation of the supernatant with the streptavidin beads at 4˚C ensured that the biotinylated proteins were bound to the beads. ACE2 was detected using human anti-rabbit polyclonal primary antibody (Cell Signaling Technology Cat # 4355S) and AlexaFluor 488 goat anti-rabbit secondary antibody. TMPRSS2 was detected using human anti-rabbit polyclonal primary antibody (NovusBio Cat # NBP2-38263) in blocking buffer and AlexaFluor 488 goat anti-rabbit secondary antibody in blocking buffer. (**d**) Three biological replicates of blebs containing TMPRSS2 proteases. (**e**) Three biological replicates of blebs derived from Vero/TMPRSS2 (JCRB # 1818) cell lines containing ACE2 receptor. (**f**) Three biological replicates of blebs derived from VeroE6 (ATCC # CRL-1586) cell lines containing ACE2 receptor.

1. **Cell bleb size characterization**

**Fig. S2.** Bleb characterization using NanoSight Nanoparticle Tracking Analyzer. As the blebs degrade, the components aggregate and form precipitates. Therefore, we used the measured particle size and count to assess bleb integrity. We measured the following samples of blebs derived from these cell lines: (**a**) Vero E6, (**b**) HEK 293T, (**c**) TMPRSS2 Vero E6, and (**d**) wtSpike-transfected HEK 293T. These plots are representative measurements taken for each of the bleb types.

1. **Cell bleb size monitoring over time**

**Fig. S3.** Cell bleb size as a function of time obtained from Dynamic Light Scattering over 21 days. The hydrodynamic radii were determined for blebs derived from the four cells lines used for this work, which include (**a**) Vero E6 cells, (**b**) HEK293-T cells, (**c**) Vero E6 TMPRSS2, and (**d**) wtSpike-transfected HEK293T cells. The sizes are depicted in the blue traces over the course of a three-week period. The average sizes remained consistent over the initial two-week period but decreased on the third week. These data aligned with the particle counts determined for each of the four bleb types (orange traces) over the same period. This was suggestive of particle degradation and was used as a guideline for experiments completed on the same set of blebs, which were confined to a two-week period. Error bars were calculated from the standard deviation (n = 3).

1. **Fluorescence Recovery After Photobleaching (FRAP) of Vero E6 cell bleb-derived hybrid supported lipid bilayers (SLBs)**

**Fig S4.** FRAP data collected from hybrid SLBs assembled using Vero E6 cell-derived blebs, done in triplicate with recovery curves for each respective sample. Each row refers to different batches of prepared PEDOT:PSS coated glass slides. Qualitative analysis of these data indicates that the bleach spot recovers suggesting we indeed have a mobile bilayer. Quantitative analysis of these data can be found in **Fig S6.**

1. **FRAP of Vero E6 TMPRSS2 cell bleb-derived hybrid SLB**

**Fig S5.** FRAP data collected from hybrid SLBs assembled using Vero E6 TMPRSS2 cell-derived blebs, done in triplicate with recovery curves for each respective sample. Like the Vero E6 data presented in Fig S4, qualitative analysis of these data indicates a mobile bilayer. Each row refers to different batches of prepared PEDOT:PSS coated glass slides. Quantitative analysis of these data can be found in **Fig S6**.

1. **Baseline correction of images acquired during FRAP experiments**

**Fig S6.** Intensity line profiles of images acquired during FRAP experiments. To account for baseline shifts during FRAP data acquisitions, we performed an intensity line plot analysis of the bleach spots before bleaching (bold black trace) and at several time points after including after ~ 1,200 s (bold blue trace- the last time point). The graphs on the left represent data after baseline corrections were (Baseline Corrected), and the ones on the right represent raw values acquired from the image analysis (Raw Values). The relative X distances (x-axis) represent the line scan across the image, with ~ 0 – 75 and ~ 175 – 250 representing the background (baseline) and 75 – 175 representing the bleach spot. The relative intensity is found on the y-axis and as can be seen on the graphs, the intensity dramatically drops and begins recovering immediately. This suggests that we indeed have a mobile bilayer. We used this data to calculate the true mobile fraction, rather than the apparent mobile fractions calculated prior to baseline correction (Fig S4, S5, S10). (**a**) Vero E6 cell bleb derived-hybrid SLBs have a mobile fraction of 0.93 and diffusion coefficient (D) of 0.18 µm^2^ s^-1^, (**b**) TMPRSS2Vero E6 cell bleb derived-hybrid SLBs have a mobile fraction of 0.92 and D of 0.16 µm^2^ s^-1^, (**c**) Spike-transfected HEK293T cell bleb derived-hybrid SLBs have a mobile fraction of 0.99 and D of 0.20 µm^2^ s^-1^.

1. **Electrical and optical readouts of VPP_Spike_ interactions with host cell bleb-derived hybrid SLB *via* early entry path**


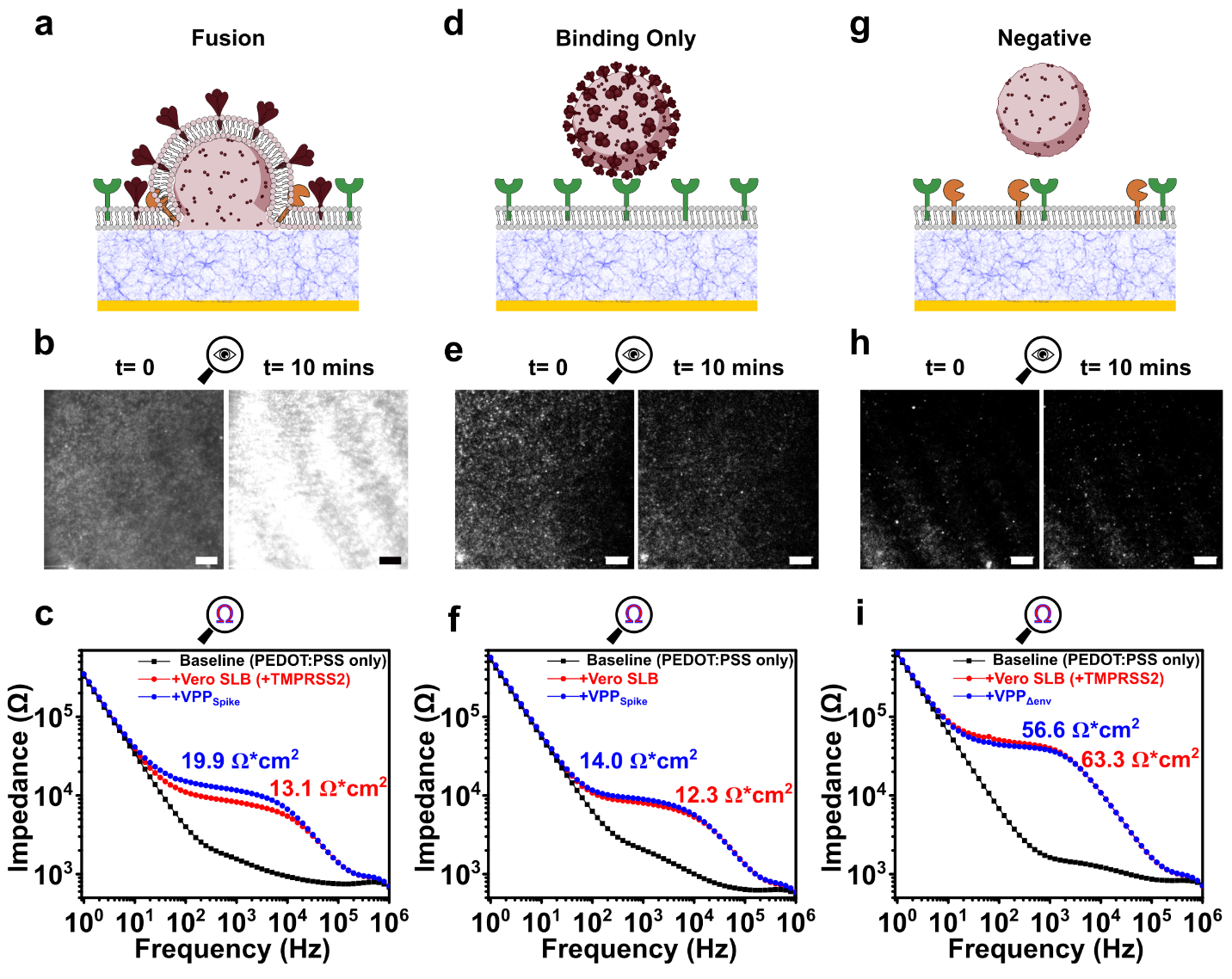


**Fig. S7.** Early entry pathway recapitulated on host cell (+ACE2, +TMPRSS2) hybrid SLB and appropriate controls. (**a**) The experimental group consisted of VPP_Spike_ and hybrid SLB containing ACE2 (green) and TMPRSS2 (yellow) where signals are characteristic of fusion events, with (**b**) showing the TIRF field of view (FOV) and changes in fluorescence after 10 minutes and (**c**) showing the changes in SLB membrane resistance in the equivalent electrical circuit scenario; (**d**) show one of the control groups where only signals coinciding to binding events are generated. This group consisted of VPP_Spike_ and SLBs containing only ACE2, (**e**) shows the TIRF data and (**f**) impedance data; (**g**) shows a negative control group where neither binding nor fusion are observed since VPP_Δenv_ were used, (**h**) shows the TIRF data and (**i**) shows impedance data; All scale bars represent 10 μm. Figure was partially created with BioRender.com.

1. **Optical confirmation of specific binding between VPP_Spike_ and ACE2 receptors**

**Fig. S8.** VPP_Spike_ interactions with hybrid SLBs with (**a**) and without (**b**) ACE2 receptors. (a) Hybrid SLB formed using blebs derived from Vero E6 cells containing ACE2 receptors. There are four representative images showing surface-bound particles; (b) Hybrid SLB formed using blebs derived from HEK 293T cells was made to evaluate nonspecific interactions with VPP_Spike_. There are minimal observable puncta in the representative images.

1. **A summary of hybrid SLB electrical properties reported in Figure 3-5**

**Table S1.** A summary of all fitted hybrid SLB electrical properties reported in Figs 3-5. Note: the two different electrode sizes refer to two different electrode configurations used, 420 µm square electrode and 420 µm diameter round electrode.

|  | **Figure 3** | | **Figure 4** | | | | **Figure 5** | |
| --- | --- | --- | --- | --- | --- | --- | --- | --- |
|  | Early pathway, Wuhan-Hu-1 | Late  pathway,  Wuhan-Hu-1 | Early pathway  Omicron BA1 | Late pathway,  Omicron BA1 | Early pathway,  Omicron BA4 | Early pathway,  Omicron BA4 | Reversed early pathway | Reversed late  pathway |
| Electrode Size  (cm^2^) | 0.001764 | 0.001385 | 0.001385 | 0.001764 | 0.001385 | 0.001385 | 0.001764 | 0.001385 |
| Initial R_SLB_  (Ω*cm^2^) | 13.09 | 9.16 | 18.70 | 13.12 | 10.60 | 10.71 | 20.29 | 24.38 |
| Initial C_SLB_  (μF/cm^2^) | 0.896 | 0.837 | 0.866 | 1.831 | 1.032 | 1.025 | 1.015 | 1.862 |
| R_SLB_ after VPP  (Ω*cm^2^) | 19.93 | - | 19.40 | - | 13.48 | - | 31.05 | - |
| C_SLB_ after VPP  (μF/cm^2^) | 0.918 | - | 0.895 | - | 0.989 | - | 1.037 | - |
| R_SLB_ after VPP and pH adjust  (Ω*cm^2^) | - | 23.28 | - | 44.28 | - | 21.20 | - | 51.68 |
| C_SLB_ after VPP and pH adjust  (μF/cm^2^) | - | 0.953 | - | 2.137 | - | 0.989 | - | 2.028 |
| R_SLB_ after cathepsin L  (Ω*cm^2^) | - | 36.71 | - | 52.21 | - | 30.20 | - | 72.18 |
| C_SLB_ after cathepsin L  (μF/cm^2^) | - | 1.018 | - | 2.143 | - | 0.989 | - | 2.476 |

1. **Electrical and optical readouts of VPP_Spike_ interacting with host cell bleb-derived hybrid SLB *via* late entry path**


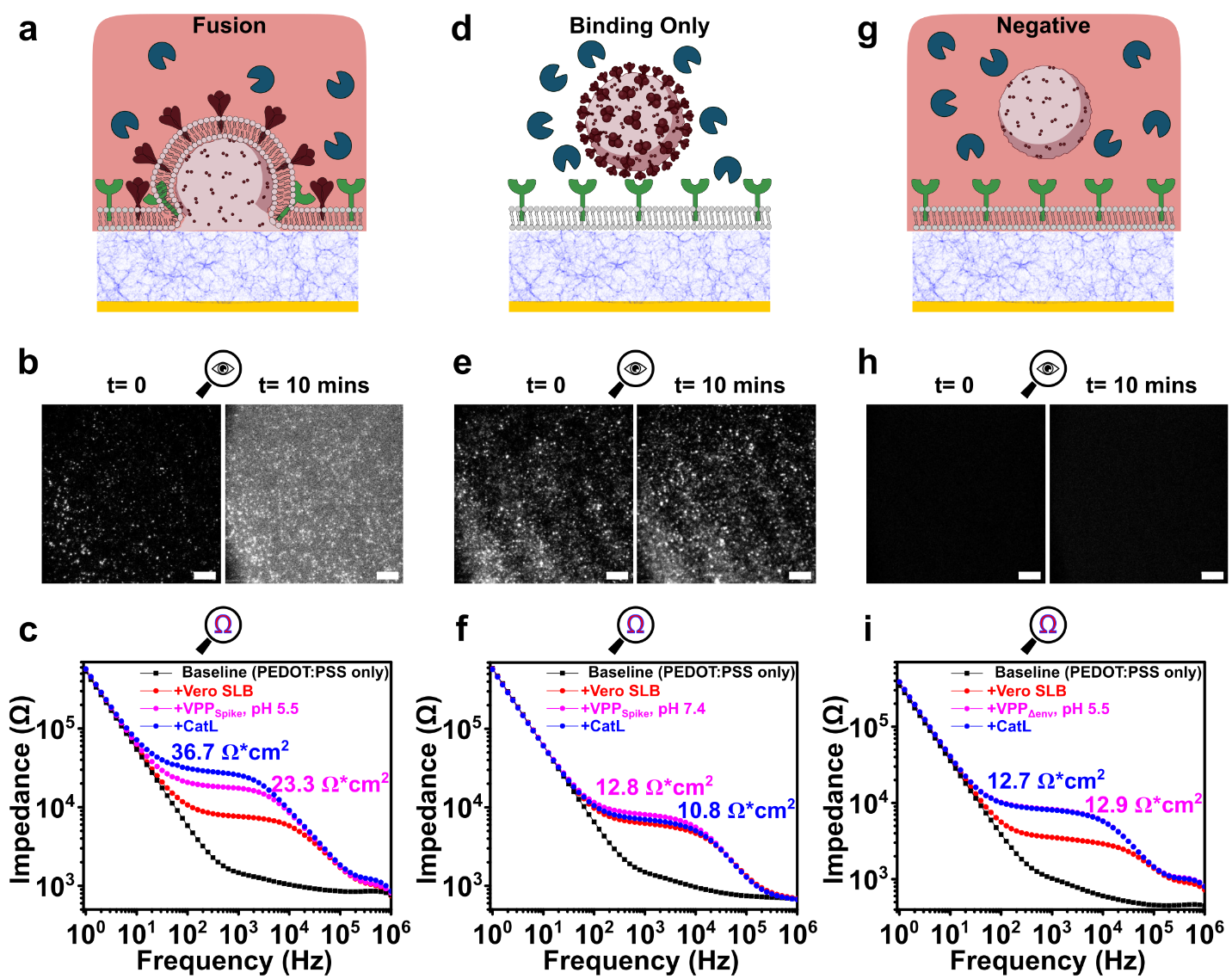


**Fig. S9.** Late entry pathway recapitulated on host cell (+ACE2) hybrid SLB. (**a**) the experimental group consisted of VPP_Spike_ and hybrid SLB containing ACE2 (green) and CatL (navy), where signals are characteristic of fusion events (note: pink = acidic environment), (**b**) shows the TIRF field of view (FOV) and changes in fluorescence after 10 minutes while (**c**) shows the SLB resistance change upon fusion after the addition of CatL at pH = 5.5; (**d**) shows one of the control groups where only signals coinciding to binding events are generated, this group consisted of VPP_Spike_ and SLBs containing ACE2 but CatL was added into a nonacidic buffer environment (pH = 7.4), (**e**) shows the TIRF data and (**f**) the impedance data; (**g**) shows a negative control group where neither binding nor fusion are observed since VPP_Δenv_ were used, (**h**) shows the TIRF data and (**i**) shows SLB resistance change upon the addition of CatL at pH = 5.5; All scale bars represent 10 μm. Figure was partially created with BioRender.com.

1. **FRAP of wtSpike-transfected HEK cell bleb-derived hybrid SLB**

**Fig S10.** FRAP data collected from hybrid SLBs assembled using wtSpike-transfected HEK 293T cell-derived blebs. Qualitative analysis of these data indicates that the bleach spot recovers suggesting we indeed have a mobile bilayer. Quantitative analysis of these data can be found in Fig S6.

1. **Sequence information for the three Spike variants (Wuhan-Hu-1 Spike, Omicron BA.1, and Omicron BA.4), Gag-Pol, and Luciferase**

**Table S2.** Sequences for the three Spike variants used for this study, including Wuhan-Hu-1 Spike, Omicron BA.1, and Omicron BA.4, Gag-Pol, and Luciferase.

| **Name** | **Sequence** |
| --- | --- |
| **Spike protein** | atgttcctgctgaccacaaagcggacaatgttcgtgtttctggtgctgctgcctctggtgagctcccagtgcgtgaacctgaccacaagaacccagctgccccctgcctataccaattccttcacacggggcgtgtactatcccgacaaggtgtttagatctagcgtgctgcactccacacaggatctgtttctgcctttcttttctaacgtgacctggttccacgccatccacgtgagcggcaccaatggcacaaagaggttcgacaatccagtgctgccctttaacgatggcgtgtacttcgcctccaccgagaagtctaacatcatccgcggctggatctttggcaccacactggacagcaagacacagtccctgctgatcgtgaacaatgccaccaacgtggtgatcaaggtgtgcgagttccagttttgtaatgatccattcctgggcgtgtactatcacaagaacaataagtcttggatggagagcgagtttcgggtgtattcctctgccaacaattgcacatttgagtacgtgtcccagcccttcctgatggacctggagggcaagcagggcaatttcaagaacctgcgggagttcgtgtttaagaatatcgatggctacttcaagatctactccaagcacaccccaatcaacctggtgagagacctgccacagggattctctgccctggagccactggtggatctgcccatcggcatcaacatcacccggtttcagacactgctggccctgcacagaagctacctgacaccaggcgacagctcctctggatggaccgcaggagctgccgcctactatgtgggctatctgcagccccggaccttcctgctgaagtacaacgagaatggcaccatcacagacgcagtggattgcgccctggaccccctgtctgagaccaagtgtacactgaagagctttaccgtggagaagggcatctatcagacaagcaatttcagggtgcagcctaccgagtccatcgtgcgctttcccaatatcacaaacctgtgcccttttggcgaggtgttcaacgccaccagattcgccagcgtgtacgcctggaataggaagcgcatctccaactgcgtggccgactattctgtgctgtacaacagcgcctccttctctacctttaagtgctatggcgtgagccccacaaagctgaatgatctgtgctttaccaacgtgtacgccgattccttcgtgatcaggggagacgaagtgaggcagatcgcaccaggacagacaggaaagatcgcagactacaattataagctgcctgacgatttcaccggctgcgtgatcgcctggaactctaacaatctggatagcaaagtgggcggcaactacaattatctgtaccggctgtttagaaagtctaatctgaagccattcgagcgggacatctccacagagatctaccaggccggctctaccccctgcaatggcgtggagggctttaactgttatttccctctgcagagctacggcttccagccaaccaacggcgtgggctatcagccctacagagtggtggtgctgtcttttgagctgctgcacgcacctgcaacagtgtgcggcccaaagaagagcaccaatctggtgaagaacaagtgcgtgaacttcaacttcaacggactgaccggcacaggcgtgctgaccgagtccaacaagaagttcctgccttttcagcagttcggcagggacatcgcagataccacagacgccgtgcgcgaccctcagaccctggagatcctggacatcacaccatgctccttcggcggcgtgtctgtgatcacaccaggcaccaatacaagcaaccaggtggccgtgctgtatcaggacgtgaattgtaccgaggtgcccgtggcaatccacgcagatcagctgacccctacatggcgggtgtactctaccggcagcaacgtgttccagacaagagccggatgcctgatcggagcagagcacgtgaacaatagctatgagtgcgacatccctatcggcgccggcatctgtgcctcctaccagacccagacaaactccccaaggagagccaggtctgtggccagccagtccatcatcgcctataccatgagcctgggcgccgagaacagcgtggcctactccaacaattctatcgccatccctaccaacttcacaatctccgtgaccacagagatcctgccagtgagcatgaccaagacatccgtggactgcacaatgtatatctgtggcgattccaccgagtgctctaacctgctgctgcagtacggctctttttgtacccagctgaatcgcgccctgacaggaatcgcagtggagcaggacaagaacacacaggaggtgttcgcccaggtgaagcagatctacaagaccccacccatcaaggactttggcggcttcaacttcagccagatcctgcccgatcctagcaagccatccaagaggtcttttatcgaggacctgctgttcaacaaggtgaccctggccgatgccggcttcatcaagcagtatggagattgcctgggagacatcgcagcccgcgacctgatctgtgcccagaagtttaatggcctgaccgtgctgcctccactgctgacagatgagatgatcgcccagtacacatctgccctgctggccggcaccatcacaagcggatggaccttcggcgcaggagccgccctgcagatcccctttgccatgcagatggcctatcggttcaacggcatcggcgtgacccagaatgtgctgtacgagaaccagaagctgatcgccaatcagtttaactccgccatcggcaagatccaggactctctgagctccacagccagcgccctgggaaagctgcaggatgtggtgaatcagaacgcccaggccctgaataccctggtgaagcagctgtctagcaacttcggcgccatctcctctgtgctgaatgacatcctgagccggctggacaaggtggaggcagaggtgcagatcgaccggctgatcacaggcagactgcagtccctgcagacctacgtgacacagcagctgatcagggcagcagagatcagggcctctgccaatctggccgccaccaagatgagcgagtgcgtgctgggacagtccaagagggtggacttttgtggcaagggctatcacctgatgagcttcccacagtccgcccctcacggcgtggtgtttctgcacgtgacctacgtgccagcccaggagaagaacttcaccacagcaccagccatctgccacgatggaaaggcacactttcctagggagggcgtgttcgtgagcaacggcacccactggtttgtgacacagcgcaatttctacgagccacagatcatcaccacagacaataccttcgtgagcggcaactgtgatgtggtgatcggcatcgtgaacaataccgtgtatgatcctctgcagccagagctggactcttttaaggaggagctggataagtacttcaagaatcacaccagccccgacgtggatctgggcgacatctctggcatcaatgccagcgtggtgaacatccagaaggagatcgacagactgaacgaggtggccaagaatctgaacgagtccctgatcgatctgcaggagctgggcaagtatgagcagtacatcaagtggccctggtatatctggctgggcttcatcgccggcctgatcgccatcgtgatggtgaccatcatgctgtgctgtatgacaagctgctgttcctgcctgaagggctgctgttcttgtggcagctgctgtaagtttgatgaggacgatagcgagcctgtgctgaagggcgtgaagctgcactacacctga |
| **BA.1** | atgttcgtgtttctggtgctgctgcctctggtgtccagccagtgtgtgaacctgaccaccagaacacagctgcctccagcctacaccaacagctttaccagaggcgtgtactaccccgacaaggtgttcagatccagcgtgctgcactctacccaggacctgttcctgcctttcttcagcaacgtgacctggttccacgtgatcagcggcaccaatggcaccaagagattcgacaaccccgtgctgcccttcaacgacggggtgtactttgccagcatcgagaagtccaacatcatccgcggctggatcttcggcaccacactggatagcaagacccagagcctgctgatcgtgaacaacgccaccaacgtggtcatcaaagtgtgcgagttccagttctgcaacgacccattcttcgaccacaagaacaacaagagctggatggaaagcgagttccgggtgtacagcagcgccaacaactgcaccttcgagtacgtgtcccagcctttcctgatggacctggaaggcaagcagggcaacttcaagaacctgcgcgagttcgtgttcaagaacatcgacggctacttcaagatctacagcaagcacacccctatcatcgtgcgcgagcctgaggatctgcctcagggcttttctgccctggaacctctggtggatctgcccatcggcatcaacatcacccggtttcagacactgctggccctgcacagaagctacctgacacctggcgatagcagcagcggatggacagctggtgccgccgcttactatgtgggctacctgcagcctagaaccttcctgctgaagtacaacgagaacggcaccatcaccgacgccgtggattgtgctctggatcccctgagcgagacaaagtgcaccctgaagtccttcaccgtggaaaagggcatctaccagaccagcaacttccgggtgcagcccaccgaatccatcgtgcggttccccaatatcaccaatctgtgccccttcgatgaggtgttcaatgccaccagattcgccagcgtgtacgcctggaaccggaagagaatcagcaactgcgtggccgactactccgtgctgtacaatctggccccattctttaccttcaagtgctacggcgtgtcccctaccaagctgaacgacctgtgcttcaccaatgtgtacgccgacagcttcgtgatccggggagatgaagtgcggcagattgcccctggacagaccggcaatatcgccgactacaactacaagctgcccgacgacttcaccggctgtgtgatcgcctggaatagcaacaagctggacagcaaggtgtccggcaactacaattacctgtaccggctgttccggaagtccaatctgaagcccttcgagcgggacatcagcaccgagatctatcaggccggcaacaagccctgtaatggcgtggccggcttcaactgctacttcccactgcggagctacagcttcagacccacatacggcgttggccaccagccttacagagtggtggtgctgtccttcgagctgctgcatgctcctgccacagtgtgcggccctaagaaaagcaccaacctcgtgaagaacaaatgcgtgaacttcaacttcaacggcctgaaaggcaccggcgtgctgaccgagagcaacaagaagttcctgccattccagcagttcggccgggacattgccgataccacagatgctgtcagagatccccagacactggaaatcctggacatcaccccttgcagcttcggcggagtgtctgtgatcacccctggcaccaacaccagcaatcaggtggcagtgctgtaccagggcgtgaactgtacagaggtgccagtggccattcacgccgatcagctgacccctacttggcgggtgtactccacaggcagcaatgtgttccagaccagagccggctgtctgattggcgccgagtatgtgaacaacagctacgagtgcgacatccccatcggagccggcatctgtgccagctaccagacacagaccaagagccacagacgggctagaagcgtggccagccagagcatcattgcctacacaatgtctctgggcgccgagaacagcgtggcctacagcaacaactctatcgctatccccaccaacttcaccatcagcgtgaccaccgagattctgcccgtgtccatgaccaagaccagcgtggactgcaccatgtacatctgcggcgattccaccgagtgctccaacctgctgctgcagtacggcagcttctgcacccagctgaagagagccctgacagggattgccgtggaacaggacaagaacacccaagaggtgttcgcccaagtgaagcagatctacaagacccctcctatcaagtacttcggcgggttcaacttctcccagatcctgccagatcctagcaagcccagcaagcggagcttcatcgaggacctgctgttcaacaaagtgacactggccgacgccggctttatcaagcagtatggcgattgcctgggcgacattgcagccagggatctgatttgcgcccagaagttcaagggcctgacagtgctgcctcctctgctgacagatgagatgatcgcccagtacacaagcgccctgctggccggcacaatcacctctggatggacatttggagccggcgctgccctgcagatcccatttgctatgcagatggcctaccggttcaacggcatcggagtgacccagaatgtgctgtacgagaaccagaagctgatcgccaaccagttcaacagcgccatcggcaagatccaggacagcctgagcagcacagcctctgctctgggcaagctgcaggacgtggtcaaccataatgcccaggcactgaacaccctggtcaagcagctgtcctccaagttcggcgccatctctagcgtgctgaatgacatcttctccaggctggacaaggtggaagccgaggtgcagatcgacagactgatcaccggaaggctgcagtccctgcagacctacgttacccagcagctgattagagccgccgagatcagagccagcgccaatctggctgccaccaagatgtctgagtgtgtgctgggccagagcaagagagtggacttttgcggcaagggctaccacctgatgagcttccctcagtctgctcctcacggcgtggtgtttctgcacgtgacatacgtgcccgctcaagagaagaatttcaccaccgctccagccatctgccacgacggcaaagcccactttcctagagaaggcgtgttcgtgtccaacggcacccattggttcgtgacccagcggaacttctacgagccccagatcatcaccaccgacaacaccttcgtgtctggcaactgcgacgtcgtgatcggcattgtgaacaataccgtgtacgaccctctgcagcccgagctggactccttcaaagaggaactggataagtactttaagaaccacacaagccccgacgtggacctgggcgatatcagcggaatcaatgccagcgtcgtgaacatccagaaagagatcgaccggctgaacgaggtggccaagaatctgaacgagagcctgatcgacctgcaagaactggggaagtacgagcagtacatcaagtggccttggtacatctggctgggctttatcgccggactgattgccatcgtgatggtcacaatcatgctgtgctgtatgaccagctgctgtagctgcctgaagggctgttgtagctgtggctcctgctgctga |
|  |  |
|  |  |
|  |  |
| **BA.4** | atgttcgtgtttctggttctcctgcccctggtgagcagccagtgtgttaatctgatcacccggacgcagagctatacaaatagcttcaccagaggcgtgtactatcctgataaggtgttcagaagcagcgtgctgcacagcacacaagatctgttcctgccttttttcagcaatgtgacctggttccacgccatcagcggcaccaacggcaccaagcggtttgacaaccctgtgctgcctttcaacgatggggtgtacttcgcctctacagagaagagcaacatcatccggggctggatcttcggcaccaccctggattctaagacccagagcttgctgatcgtgaacaatgctaccaacgtggtgatcaaagtgtgtgaattccagttctgcaacgacccttttctggatgtgtactaccacaagaacaacaagtcttggatggaaagcgagttcagagtgtattcatctgccaacaactgcaccttcgagtacgtgtctcaacctttcctgatggacctggaaggcaagcagggcaacttcaagaaccttagagaattcgtgttcaagaacatcgacggctacttcaagatctactctaagcacacacccatcaacctgggacgggacctcccccaaggcttcagcgcccttgagcccctggtggacctgcctatcggcatcaacatcacccggttccagaccctgctggctctgcatagaagctacctgaccccaggcgactctagcagcggctggaccgccggagccgccgcctactatgtgggctacctgcaacctagaactttcctgctcaagtacaatgagaatggcaccatcaccgacgccgtcgactgcgccctggatcctctgagcgagacaaagtgcacactgaaaagtttcaccgtggaaaaaggcatctatcagacctctaactttagagtgcaacctaccgagtcaatcgtgcggttccctaacatcaccaatctgtgtccttttgacgaggtgttcaacgctacaaggttcgccagcgtgtacgcctggaaccggaaacggatctccaattgcgtggccgactacagcgtgctgtacaacttcgcccctttctttgccttcaagtgctacggagtgtctccaacaaagctgaatgacctgtgcttcaccaatgtgtacgcagacagcttcgtgatcagaggcaacgaggtgagccaaatcgcccccggccagacaggaaacattgccgattacaactacaagctacctgacgatttcaccggctgcgtgatagcctggaactctaacaagctggatagcaaggtgggaggaaactacaactacagatacagactgttcagaaagtctaacctgaaaccttttgaaagagatatctctaccgagatctaccaggccggtaacaaaccgtgcaacggagtggccggcgtgaactgctactttccactgcagagctacggcttcagaccaacctacggcgttggccaccagccttaccgggtggtggtgctgagcttcgagctgctgcacgcccctgccaccgtgtgcggacctaagaaatcgacaaacctggtgaaaaacaagtgcgtgaattttaacttcaacggcctgacaggcacaggcgtgctgacagaaagtaacaaaaagttcctgcccttccagcagttcggaagagatatcgccgacaccacagatgccgtgcgggacccccagaccttggagatcctggacatcacaccttgtagctttggcggcgtgagcgtcataaccccaggcacaaataccagcaaccaggtggccgtgctgtaccagggcgtgaactgcaccgaggttcccgtggctattcacgccgaccagctgacacctacatggcgggtgtacagcaccggctctaacgtgttccagaccagagccggctgcctgatcggagctgagtatgtgaacaacagctatgaatgtgacatccctatcggagctggcatttgtgccagctaccagacccagacgaaaagccaccgcagagccagaagcgtcgccagccagagtatcatcgcctacacgatgagcctgggcgcagagaactccgtggcctactccaacaactctatcgccatccccacaaacttcactatctctgtcacaaccgaaattctgcccgtgagtatgaccaagaccagcgtcgactgcaccatgtacatctgtggcgacagcacagagtgtagcaacctgctgctgcagtacgggagcttttgtacacagctgaagagagccctgacgggcatcgcagttgaacaggacaagaatacccaggaggtgttcgcccaggtgaagcagatttacaagacccctcctattaagtactttggcggattcaacttcagccagatcctgcctgaccctagcaagccttcgaagcggagcttcatcgaggaccttctctttaacaaagtgacgctggccgacgccggcttcatcaagcagtacggcgactgcctgggcgacattgcagctagagacctgatctgcgcccagaagtttaacggcctgaccgtgctgcctcctctgctgaccgacgaaatgatcgctcaatacacaagcgccttactggccggcaccatcacttccggatggacattcggcgccggcgccgccctgcagattcctttcgctatgcagatggcttatcgcttcaacggcatcggcgtgacccagaacgtgctatacgagaaccagaagctgatcgccaatcagtttaactcagctattggcaagatccaggattccctgtcctctacagccagcgccctgggtaaactgcaagatgtggtgaaccacaacgctcaggccctgaacacactggtgaagcagctgagctccaagtttggcgccatcagctctgtcctgaatgacattctgagcagactggacaaggtcgaagccgaggtgcagatcgacagactgatcaccggcaggctgcaaagtctgcagacatacgtgacgcagcagctgattagagccgccgaaatccgggcatctgcaaatctggctgccacaaagatgtccgagtgcgtgctggggcagagcaagagagtcgacttctgcggcaaaggctaccacctgatgagcttcccccagtctgccccgcacggcgtggttttcctgcatgtgacctacgtgcccgctcaggagaaaaatttcaccaccgcccctgccatttgccacgacggaaaggcccacttccccagagagggcgttttcgtgagcaacggcacccactggttcgtgacacagagaaacttctacgagcctcagattatcaccaccgataacacattcgtgtccggcaactgcgacgtggtgatcggcatcgtgaataacacagtgtacgaccctctgcagcccgagctggatagcttcaaggaagagctggacaaatacttcaagaaccacaccagccccgatgtggacctgggcgatatctctggaatcaacgccagcgtggtgaacatccagaaggaaatcgatagactcaacgaggtggccaaaaacctgaacgagagcctgatcgacctccaagagctgggcaagtacgagcagtatatcaagtggccttggtacatctggctgggcttcatcgccggactgatcgctatcgtgatggtgaccatcatgctgtgctgtatgacttcttgctgcagctgtctgaagggttgttgctcttgcggctcctgctgctga |
| **Gag/Pol** | atgggccaggctgttaccacccccttaagtttgactttagaccactggaaggatgtcgaacggacagcccacaacctgtcggtagaggttagaaaaaggcgctgggttacattctgctctgcagaatggccaaccttcaacgtcggatggccacgagacggcacttttaacccagacattattacacaggttaagatcaaggtcttctcacctggcccacatggacatccggatcaggtcccctacatcgtgacctgggaagctatagcagtagacccccctccctgggtcagacccttcgtgcaccctaaacctcccctctctcttcccccttcagccccctctctcccacctgaacccccactctcgaccccgccccagtcctccctctatccggctctcacttctcctttaaacaccaaacctaggcctcaagtccttcctgatagcggaggaccactcattgatctactcacggaggaccctccgccttaccgggacccagggccaccctctcctgacgggaacggcgatagcggagaagtggcccctacagaaggagcccctgacccttccccaatggtatcccgcctgcggggaagaaaagaaccccccgtggcggattctactacctctcaggcgttcccccttcgcctgggagggaatggacagtatcaatactggccattttcctcctctgacctctataactggaaaaataacaacccctctttctccgaggacccagctaaattgacagctttgatcgagtccgttctccttactcatcagcccacttgggatgactgccaacagctattagggaccctgctgacgggagaagaaaaacagcgagtgctcctagaggcccgaaaggcggttcgaggggaggacggacgcccaactcagctgcccaatgacattaatgatgcttttcccttggaacgtcccgactgggactacaacacccaacgaggtaggaaccacctagtccactatcgccagttgctcctagcgggtctccaaaacgcgggcagaagccccaccaatttggccaaggtaaaagggataacccagggacctaatgagtctccctcagcctttttagagagactcaaggaggcctatcgcagatacactccttatgaccctgaggacccagggcaagaaaccaatgtggccatgtcattcatctggcagtccgccccggatatcgggcgaaagttagagcggttagaagatttgaagagtaagaccttaggagacttagtgagggaagctgaaaagatctttaataaacgagaaaccccggaagaaagagaggaacgtattaggagagaaacagaggaaaaggaagaacgccgtagggcagaggatgtgcagagagagaaggagagggaccgcagaagacatagagaaatgagtaagttgctggctactgtcgttagcgggcagagacaggatagacagggaggagagcgaaggaggccccaactcgaccacgaccagtgtgcctactgcaaagaaaagggacattgggctagagattgccccaagaagccaagaggaccccggggaccacgaccccaggcctccctcctgaccttagacgattagggaggtcagggtcaggagcccccccctgaacccaggataaccctcagagtcggggggcaacccgtcaccttcctagtggatactggggcccaacactccgtgctgacccaaaatcctggacccctaagtgacaagtctgcctgggtccaaggggctactggagggaagcggtatcgctggaccacggatcgccgagtgcacctagccaccggtaaggtcacccattctttcctccatgtaccagattgcccctatcctctgctaggaagagatttgctgactaaactaaaagcccaaattcactttgagggatcaggagctcaggttgtgggaccaatgggacagcccctgcaagtgctgaccctaaacatagaagatgagtatcggctacatgagacctcaaaagggccagatgtgcctctagggtccacatggctctctgattttccccaggcctgggcagaaaccgggggcatggggctggccgttcgccaagctcctctgatcatacctctgaaggcaacctctacccccgtgtccataaaacaataccccatgtcacaagaagccagactggggatcaagccccacatacagagactgctggatcagggaattctggtaccctgccagtccccctggaacacgcccctgctacccgttaagaaaccggggactaatgattataggcctgtccaggatctgagagaagtcaacaagcgggtggaagacatccaccccaccgtgcccaacccttacaacctcttgagcgggctcccaccgtcccaccagtggtacactgtgcttgacttaaaagatgcttttttctgcctgagactccaccccaccagtcagtctctcttcgcctttgagtggagagatccagagatgggaatctcaggacaattaacctggaccagactcccgcagggtttcaaaaacagtcccaccctgtttgatgaagccctgcacagggacctcgcagacttccggatccagcacccagacctgattctgctccagtatgtagatgacttactgctggccgccacttctgagcttgactgtcaacaaggtacgcgggccctgttacaaaccctaggggacctcggatatcgggcctcggccaagaaagcccaaatttgccagaaacaggtcaagtatctggggtatcttctaaaagagggtcagagatggctgactgaggccagaaaagagactgtgatggggcagcctactccgaagacccctcgacaactaagggagttcctagggacggcaggcttctgtcgcctctggatccctgggtttgcagaaatggcagcccccttgtaccctctcaccaaaacggggactctgtttgagtggggcccagaccagcaaaaggcctaccaagagatcaagcaggctctcttaactgcccctgccctgggattgccagacttgactaagcccttcgaactttttgttgacgagaagcagggctacgccaaaggtgtcctaacgcaaaaactggggccttggcgtcggccggtggcctacctgtccaaaaagctagacccagtggcagctgggtggcccccttgcctacggatggtagcagccatcgccgttctgaccaaagacgctggcaagctcaccatgggacagccactagtcattctggccccccatgcagtagaggcactagttaagcaaccccctgatcgctggctctccaacgcccgaatgacccactaccaggctctgcttctggacacggaccgagtccagttcggaccaatagtggccctaaacccagctacgctgctccctctacctgaggaggggctgcaacatgactgccttgacatcttggctgaagcccacggaactagaccagatcttacggaccagcctctcccagacgctgaccacacctggtacacagatgggagcagcttcctgcaagaggggcagcgcaaggccggagcagcagtaaccaccgagaccgaggtagtctgggccaaagcactgccagccgggacatcggcccaaagagctgagttgatagcgctcacccaagccttaaaaatggcagaaggtaagaagctgaatgtttacaccgatagccgttatgcttttgccactgcccatattcacggagaaatatatagaaggcgcgggttgctcacatcagaaggaaaagaaatcaaaaataaggacgagatcttggccctactgaaggctctcttcctgcccaaaagacttagcataattcattgcccgggacatcagaagggaaaccgcgcggaggcaaggggcaacaggatggccgaccaagcggcccgagaagtagccactagagaaactccagagacttccacacttctgatagaaaattcagccccctatactcatgaacattttcactatacggtgactgacataaaagatctgactaaactaggggccacttatgacgatgcaaagaagtgttgggtttatcagggaaagcctgtaatgcctgatcaattcacctttgaactattagattttcttcatcaattgacccacctcagtttctcaaaaacaaaggctcttctagaaaggaactactgtccttattacatgctgaaccgggatcgaacgctcaaagacatcactgagacttgccaagcctgtgcacaggtcaatgccagcaagtctgccgtcaaacaagggactagagttcgagggcaccgacccggcacccactgggaaattgatttcactgaggtaaaacctggcctgtatgggtataaatatcttttagttttcatagacactttctctggatgggtagaagctttcccaaccaagaaagaaactgccaaagttgtaaccaagaagctactagaagaaatcttccccagattcggcatgccacaggtattgggaaccgacaatgggcctgccttcgtctccaaggtaagtcagacagtagccgatttactgggggttgattggaaactacattgtgcttacagaccccagagttcaggtcaggtagaaagaatgaataggacaatcaaggagactttaactaaattgacgcttgcaactggctctagggactgggtgctcctgcttcccctagccctgtatcgagcccgcaacacgccgggcccccatggtctcaccccatatgaaatcttatatggggcacccccgccccttgtaaacttccctgatcctgacatggcaaaggttactcataacccctctctccaagcccatttacaggcactctacctggtccagcacgaagtctggagaccgttggcggcagcttaccaagaacaactggaccggccggtagtgcctcaccctttccgagtcggtgacacagtgtgggtccgcagacaccaaactaaaaatctagaaccccgctggaaaggaccttataccgtcctactgactacccccaccgctctcaaagtggacggcattgcagcgtggatccacgctgcccacgtaaaggctgccgacaccaggattgagccaccatcggaatcgacatggcgtgttcaacgctctcaaaatcccctaaagataagattgacccgcgggacctcctaa |
| **Luciferase** | atggaagacgccaaaaacataaagaaaggcccggcgccattctatccgctggaagatggaaccgctggagagcaactgcataaggctatgaagagatacgccctggttcctggaacaattgcttttacagatgcacatatcgaggtggacatcacttacgctgagtacttcgaaatgtccgttcggttggcagaagctatgaaacgatatgggctgaatacaaatcacagaatcgtcgtatgcagtgaaaactctcttcaattctttatgccggtgttgggcgcgttatttatcggagttgcagttgcgcccgcgaacgacatttataatgaacgtgaattgctcaacagtatgggcatttcgcagcctaccgtggtgttcgtttccaaaaaggggttgcaaaaaattttgaacgtgcaaaaaaagctcccaatcatccaaaaaattattatcatggattctaaaacggattaccagggatttcagtcgatgtacacgttcgtcacatctcatctacctcccggttttaatgaatacgattttgtgccagagtccttcgatagggacaagacaattgcactgatcatgaactcctctggatctactggtctgcctaaaggtgtcgctctgcctcatagaactgcctgcgtgagattctcgcatgccagagatcctatttttggcaatcaaatcattccggatactgcgattttaagtgttgttccattccatcacggttttggaatgtttactacactcggatatttgatatgtggatttcgagtcgtcttaatgtatagatttgaagaagagctgtttctgaggagccttcaggattacaagattcaaagtgcgctgctggtgccaaccctattctccttcttcgccaaaagcactctgattgacaaatacgatttatctaatttacacgaaattgcttctggtggcgctcccctctctaaggaagtcggggaagcggttgccaagaggttccatctgccaggtatcaggcaaggatatgggctcactgagactacatcagctattctgattacacccgagggggatgataaaccgggcgcggtcggtaaagttgttccattttttgaagcgaaggttgtggatctggataccgggaaaacgctgggcgttaatcaaagaggcgaactgtgtgtgagaggtcctatgattatgtccggttatgtaaacaatccggaagcgaccaacgccttgattgacaaggatggatggctacattctggagacatagcttactgggacgaagacgaacacttcttcatcgttgaccgcctgaagtctctgattaagtacaaaggctatcaggtggctcccgctgaattggaatccatcttgctccaacaccccaacatcttcgacgcaggtgtcgcaggtcttcccgacgatgacgccggtgaacttcccgccgccgttgttgttttggagcacggaaagacgatgacggaaaaagagatcgtggattacgtcgccagtcaagtaacaaccgcgaaaaagttgcgcggaggagttgtgtttgtggacgaagtaccgaaaggtcttaccggaaaactcgacgcaagaaaaatcagagagatcctcataaaggccaagaagggcggaaagatcgccgtgtaa |

1. **Optimization of R18 labeling of VPP_Spike_ to achieve semi-quenched state for optical characterization of VPP-SLB interactions**

**Fig S11.** Optimization of R18 labeling of SARS-CoV-2 pseudoparticles to achieve semi-quenched labeling state for optical imaging of fusion. ~ 10^9^ Wuhan-Hu-1 Spike pseudoparticles/mL were labeled with two different R18 concentrations: 500 ng (orange) versus 5,000 ng (blue) and the samples were placed in a fluorimeter to compare their fluorescent traces. **a** represents the first 100 seconds of the trace and is therefore the background signal; **b** indicates when the pseudoparticles were added at t =100 seconds and shows the corresponding increase in fluorescence. At t = 200 seconds, 50 μLs of a 10 % (v/v) Triton-X 100 solution were added to the 1 mL pseudoparticle samples. The results indicated that labeling with 500 ng of R18 did not result in pseudoparticles being in a quenched state, as evidenced by the small increase in signal upon the addition of detergent (**c**). Conversely, the fluorophores were in a semi-quenched state when 10-fold more R18 was used to label the pseudoparticles (**d**). This is indicated by the dramatic increase in signal upon Triton-X 100 addition, in comparison to the low but detectable signal prior to detergent addition. The dip in fluorescence observed at t = 500 seconds was due to additional detergent being added to confirm dequenching was complete (black arrow).

1. **Impacts of ethylene glycol and acidic buffer on PEDOT:PSS electrode electrical properties**


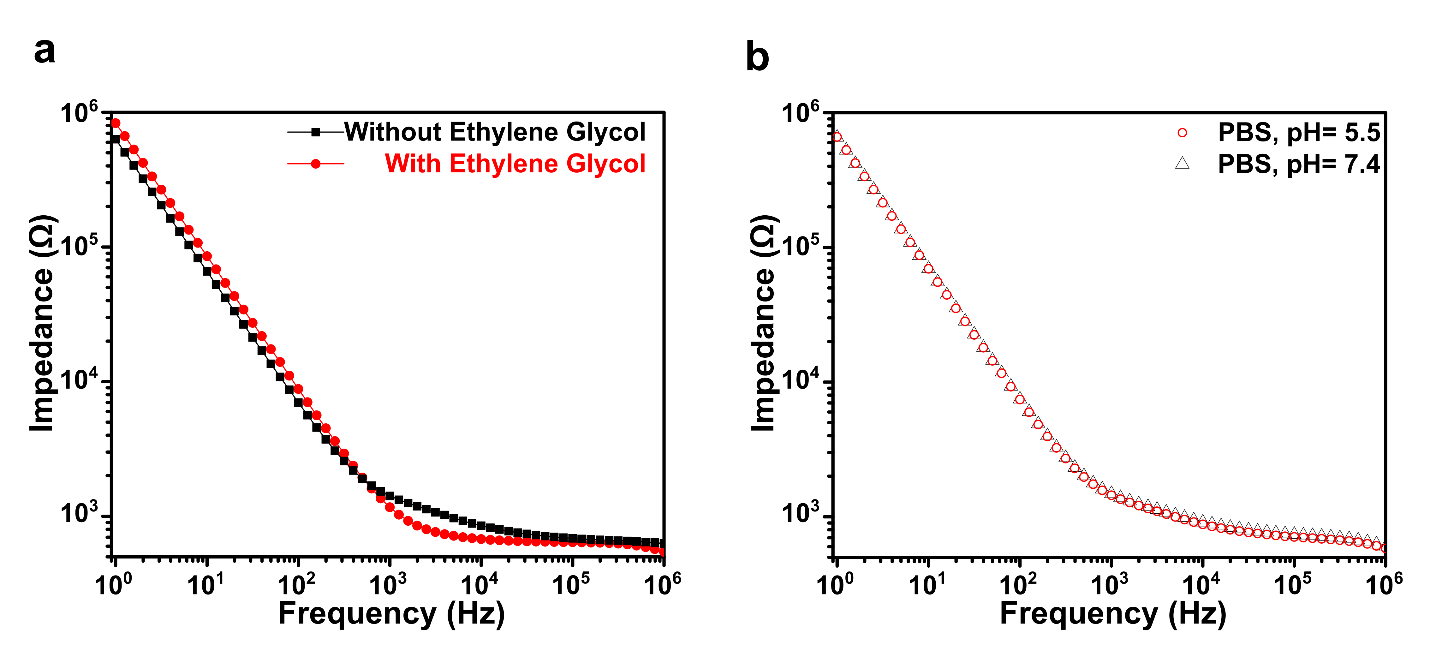


**Figure S12.** The impact of ethylene glycol and acidic PBS buffer on PEDOT:PSS electrode electrical properties. **(a)** The addition of ethylene glycol improves the electrical conductivity of PEDOT:PSS film, resulting in an ideal “L” shape curve in red. However, without the addition of this component in our formulation a more gradual transition between the capacitive region at low frequency and the resistive region at high frequency is observed at the middle frequencies as shown in black. Ethylene glycol additive was not used in this work, so our baselines typically exhibit this non-ohmic behavior, which is masked when the SLB is formed; (**b**) The change to acidic PBS buffer (pH = 5.5, plotted in red circle) from pH 7.4 (plotted in black triangle) does not change PEDOT:PSS electrode electrical properties, indicating SLB impedance shift during late entry pathway in Figure 3, 4 and 5 do not result from an electrical property change of PEDOT:PSS electrode.
